# Maternal and Neonatal Outcomes of Iron Deficiency Anemia: A Retrospective Cohort Study

**DOI:** 10.7759/cureus.51365

**Published:** 2023-12-30

**Authors:** Sanaz Safarzadeh, Farzaneh Banihashemi, Farideh Montazeri, Nasibeh Roozbeh, Fatemeh Darsareh

**Affiliations:** 1 Mother and Child Welfare Research Center, Hormozgan University of Medical Sciences, Bandar Abbas, IRN

**Keywords:** neonatal outcomes, maternal outcomes, childbirth, pregnancy, iron deficiency anemia, anemia

## Abstract

Introduction: Understanding the outcomes of anemia in pregnancy is critical. Since no study has been conducted regarding the maternal and neonatal outcomes of iron-deficiency anemia in Hormozgan province of Iran, this study aims to assess the maternal and neonatal outcomes of iron-deficiency anemia in women who gave birth in Hormozgan province from January 2020 to January 2022.

Methods: We retrospectively assessed all singleton pregnant women who gave birth at a tertiary hospital in Bandar Abbas, Hormozgan province, Iran, for two years. We divided all women into iron-deficiency anemic and non-iron-deficiency anemic women. Iron-deficiency anemia was defined as hemoglobin less than 10.5 mg/dl at the time of admission without any other hemoglobinopathy, such as sickle cell anemia or thalassemia. Using electronic patient records, data were extracted from the Iranian Maternal and Neonatal Network (IMaN Net), a valid national system. Since the information of birth under 24 weeks of gestation is not recorded in this system, we excluded all deliveries under 24 weeks of gestation. The outcome measures of the study were demographic factors (age, education, residency place, access to prenatal care, smoking), obstetrical factors (parity, labor induction, fetal presentation, mode of delivery), and maternal and neonatal outcomes (the incidence of preeclampsia, gestational diabetes, placenta abruption, postpartum hemorrhage, maternal need for blood transfusion, maternal need for intensive care unit, preterm birth, low birth weight, intrauterine growth retardation, birth asphyxia, stillbirth, and neonatal intensive care admission). Chi-square tests were used to compare differences between iron-deficiency anemic and non-iron-deficiency anemic women. Logistic regression models were used to assess the effect of iron-deficiency anemia on maternal and neonatal outcomes. The result was presented as odds ratio (OR) or adjusted odds ratio (aOR) after adjusting for covariates and a 95% confidence interval (CI).

Results: The incidence of iron-deficiency anemia was 2.97%. Education and residency were among the demographic factors that differed significantly between groups. Iron-deficiency anemia was more frequent in those with higher education and women who lived in rural areas. In terms of obstetrical factors, method of delivery was the only significantly different factor between groups. Iron-deficiency anemic mothers had substantially more instrumental deliveries than non-iron-deficiency anemic mothers (4.3% vs. 0.8%), while the incidence of cesarean section was lower. Based on logistic regression in terms of maternal and neonatal outcomes, iron-deficiency anemic women had a substantially higher risk of the need for maternal blood transfusion (aOR: 6.54, 95%CI: 4.72-8.15), postpartum hemorrhage (aOR: 1.54, 95%CI: 0.71-2.11), preterm birth (aOR: 0.98, 95%CI: 0.45-1.13), low birth weight (aOR: 1.04, 95%CI: 0.78-2.01), intrauterine growth retardation (aOR: 1.30, 95%CI: 0.99-2.10), and neonatal intensive care admission (aOR: 1.06, 95%CI: p.52-2.72), after adjusting for educational level, residency place, and method of delivery.

Conclusions: Despite the higher incidence of postpartum hemorrhage and maternal blood transfusion, we found no increase in maternal intensive care unit admission risk. Regarding neonatal outcomes, iron-deficiency anemia was linked to preterm birth, low birth weight, intrauterine growth retardation, and neonatal intensive care admission.

## Introduction

Even though anemia appears to influence women in both high- and low/middle-income countries, the major burden of illness is found in low/middle-income nations. According to the latest WHO data, 37% of pregnant women and 30% of women at reproductive age worldwide are anemic [1]. Anemia in pregnancy is defined as a serum hemoglobin level of 11.0 mg/dl in the first trimester, 10.5 mg/dl in the second and third trimesters, and 10.0 mg/dl after delivery [2]. The prevalence of anemia during pregnancy ranges from between 2% and 26%, depending upon the population studied [3-5]. The two most common causes of anemia in pregnancy are iron deficiency and acute blood loss, with iron deficiency anemia considered the most common pathologic cause. Other anemia-producing disorders like malaria or hemoglobinopathies are less common in pregnancy [2,6]. The prevalence of iron deficiency anemia in pregnant women in Iran has been reported to be 15% [7].

Since anemia may be a common condition influencing pregnancy, particularly in low/middle-income nations, understanding the effect of different levels of anemia on pregnant women and their newborns is vital. Several primary research studies have been conducted to determine maternal anemia’s potential effects on adverse maternal and neonatal outcomes, such as preterm birth, low birth weight newborns [8], and postpartum hemorrhage [9]. However, the findings across the studies are inconsistent, making it difficult to develop evidence-based policies to mitigate these negative consequences [10,11]. In addition, most studies did not determine whether the anemia was due to iron deficiency or hemoglobinopathy. For example a recent systematic review and meta-analysis showed an increased risk of preterm birth with maternal anemia, however, it was not clear whether the anemia was due to iron deficiency or hemoglobinopathy [11]. Considering the importance of anemia in pregnant women and since no study has been conducted regarding the maternal and neonatal outcome of iron-deficiency anemia in Hormozgan province of Iran, this study aims to assess the consequences of iron-deficiency anemia on mothers living in Hormozgan province.

## Materials and methods

This study complied with the Declaration of Helsinki and was performed according to ethics committee approval. The Ethics and Research Committee of the Hormozgan University of Medical Sciences approved the study (HUMS.RC.1402). The records of all patients who provided informed consent for using their data for research purposes were analyzed. Statistical analysis was performed with patient anonymity following ethics committee regulations.

The objective of the study was to assess the maternal and neonatal outcomes of women with iron-deficiency anemia. We retrospectively assessed all singleton pregnant women who gave birth at a tertiary hospital in Bandar Abbas, Hormozgan province, Iran, for two years from January 2020 to January 2022. We divided all women into iron-deficiency anemic and non-iron-deficiency anemic women. Iron-deficiency anemia was defined as hemoglobin less than 10.5 mg/dl at the time of admission without any other hemoglobinopathy, such as sickle cell anemia or thalassemia. Using electronic patient records, data were extracted by trained collectors from the Iranian Maternal and Neonatal Network (IMaN Net), a valid national system. Since the information of birth under 24 weeks of gestation is not recorded in this system, we excluded all deliveries under 24 weeks of gestation.

The outcome variables of our study were preeclampsia, gestational diabetes, placenta abruption, postpartum hemorrhage, maternal need for blood transfusion, maternal need for an intensive care unit, preterm birth, low birth weight, intrauterine growth restriction, birth asphyxia, stillbirth, and neonatal intensive care admission.

The exposure variables were the presence or absence of anemia. Potential confounding factors considered were demographic factors (age, education, residency place, access to prenatal care, and smoking) and obstetrical factors (parity, labor induction, fetal presentation, and method of delivery).

The IBM Statistical Package for the Social Sciences Statistics, version 19 (IBM Corp, Armonk, NY, USA), was used to analyze the data. Categorical variables are presented as numbers and frequencies (%). Chi-square tests were used to compare differences between iron-deficiency anemic and non-iron-deficiency anemic women. Logistic regression models were used to assess the effect of anemia on adverse maternal and neonatal outcomes. The result was presented as odds ratio (OR) or adjusted odds ratio (aOR) after adjusting for covariates (educational level, living residency, and method of delivery) and a 95% confidence interval (CI). We selected all confounding factors with p-values less than 0.05 for adjusting. P < 0.05 was considered statistically significant; all statistical tests were two-tailed.

## Results

During the study period, 8631 women without iron-deficiency anemia and 257 women with iron-deficiency anemia gave birth at our center. The frequency of iron-deficiency anemia was 2.89%. Table 1 compares the demographic characteristics of women with or without iron-deficiency anemia. Educational level and residency place were among the demographic factors that differed significantly between iron-deficiency anemic and non-iron-deficiency anemic women. Iron-deficiency anemia was more frequent in those with higher education and women who lived in rural areas (p<0.01).

**Table 1:** Maternal characteristics of women diagnosed with or without iron-deficiency anemia Data are presented as n (%).

Demographic characteristics	Non-iron-deficiency anemic (n=8631)	Iron-deficiency anemic (n=257)	P-value
Age (Years)			0.927
13-19	167 (1.9)	6 (2.3)	
20-34	7061 (81.8)	207 (80.5)	
35 and above	1402 (16.2)	44 (17.1)	
Educational level			0.001
Illiterate	547 (6.3)	10 (3.9)	
Elementary	2677 (31.0)	53 (20.6)	
High school/Diploma	3954 (45.8)	135 (52.5)	
Advanced	1451 (16.8)	59 (23.0)	
Residency place			0.001
Urban	5756 (66.7)	145 (56.4)	
Rural	2875 (33.3)	112 (43.6)	
Access to prenatal care			0.134
Yes	8380 (97.1)	253 (98.4)	
No	251 (2.9)	4 (1.6)	
Smoking			0.157
Yes	8564 (99.2)	252 (98.1)	
No	66 (0.8)	5 (1.9)	

Obstetrical factors were compared between iron-deficiency anemic and non-iron-deficiency anemic women, as shown in Table 2. Method of delivery was the only significantly different factor between iron-deficiency anemic and non-iron-deficiency anemic women. Iron-deficiency anemic mothers had substantially more instrumental deliveries (all by vacuum) than non-iron-deficiency anemic mothers (4.3% vs. 0.8%), while the incidence of cesarean section was lower.

**Table 2:** Obstetric characteristics of women diagnosed with or without iron-deficiency anemia Data are presented as n (%).

Variables	Non-iron-deficiency anemic (n=8631)	Iron-deficiency anemic (n=257)	P-value
Parity			0.527
Primiparous	2430 (28.2)	77 (30.0)	
Multiparous (2-5)	6201 (71.8)	180 (70.0)	
Labor induction			0.071
No	6633 (76.9)	186 (72.4)	
Yes	1998 (23.1)	71 (27.6)	
Fetal presentation			0.525
Cephalic	8267 (95.8)	249 (96.9)	
Breech/ Transverse	364 (4.2)	8 (3.1)	
Method of delivery			<0.001
Normal vaginal delivery	5659 (65.6)	189 (73.5)	
Instrumental delivery	72 (0.8)	11 (4.3)	
Cesarean section	2900 (33.6)	57 (22.2)	

Maternal and neonatal outcomes of anemia are illustrated in Table 3. Maternal blood transfusion, preterm birth, low birth weight, intrauterine growth retardation, need for neonatal resuscitation, and maternal intensive care unit admission was more common in iron-deficiency anemic women. 

**Table 3:** Maternal and neonatal outcomes of women diagnosed with or without iron-deficiency anemia Data are presented as n (%).

Outcome	Non-iron-deficiency anemic (n=8631)	Iron-deficiency anemic (n=257)	P-value
Preeclampsia			0.302
No	8066 (93.5)	245 (95.3)	
Yes	565 (6.5)	12 (4.7)	
Gestational diabetes			0.069
No	7311 (84.7)	229 (89.1)	
Yes	1320 (15.3)	28 (10.9)	
Placenta abruption			0.999
No	8354 (96.8)	249 (96.9)	
Yes	277 (3.2)	8 (3.1)	
Stillbirth			0.060
No	8569 (99.3)	254 (98.8)	
Yes	62 (0.7)	3 (1.2)	
Postpartum hemorrhage			<0.001
No	7904 (91.6)	225 (87.5)	
Yes	727 (8.4)	32 (12.5)	
Maternal blood transfusion			<0.001
No	8510 (98.6)	215 (83.7)	
Yes	121 (1.4)	42 (16.3)	
Maternal intensive care unit admission			0.137
No	8539 (98.9)	250 (97.3)	
Yes	92 (1.1)	7 (2.7)	
Low birth weight			<0.001
No	7459 (86.4)	211 (82.1)	
Yes	1172 (13.6)	46 (17.9)	
Intrauterine growth retardation			<0.01
No	8356 (96.8)	243 (94.6)	
Yes	275 (3.2)	14 (5.4)	
Preterm Birth			<0.001
No	7429 (86.1)	213 (82.9)	
Yes	1202 (13.9)	44 (17.1)	
Newborn asphyxia			0.389
No	8546 (99.0)	252 (98.1)	
Yes	85 (1.0)	5 (1.9)	
Neonatal intensive care unit admission			<0.01
No	6901 (79.9)	195 (75.9)	
Yes	1730 (20.1)	62 (24.1)	

Table 4 represents the impact of iron-deficiency anemia on adverse events of pregnancy and childbirth based on logistic regression analysis. We set non-iron-deficiency anemic women as a reference group. Iron-deficiency anemic women had a significantly higher risk of the need for maternal blood transfusion (aOR: 6.04, 95%CI: 4.72-8.15), postpartum hemorrhage (aOR: 1.02, 95%CI: 0.71-2.11), preterm birth (aOR: 0.98, 95%CI: 0.45-1.13), low birth weight (aOR: 1.04, 95%CI: 0.78-2.01), intrauterine growth retardation (aOR: 1.30, 95%CI: 0.99-2.10), and neonatal intensive care admission (aOR: 1.06, 95%CI: 0.52-2.72), after adjusting for educational level, residency place, and method of delivery.

**Table 4:** Logistic regression analyses of adverse pregnancy and childbirth outcomes of women diagnosed with iron-deficiency anemia OR: Odds Ratio; aOR: adjusted Odds Ratio

Outcome	OR (95% CI)	P-value	a*OR (95% CI)	P-value
Need for maternal blood transfusion	13.73 (5.76-18.11)	< 0.001	6.04 (4.72-8.15)	< 0.001
Postpartum hemorrhage	1.54 (1.08-3.78)	< 0.001	1.02 (0.71-2.11)	< 0.001
Preterm labor	1.27 (0.99-3.13)	< 0.01	0.98 (0.45-1.13)	< 0.01
Low birth weight	1.38 (1.18-2.91)	< 0.01	1.04 (0.78-2.01)	< 0.01
Intrauterine growth retardation	1.75(0.86-4.08)	< 0.01	1.30 (0.99-2.10)	< 0.01
Neonatal intensive care unit admission	1.26 (1.05-2.93)	< 0.01	1.06 (0.52-2.72)	< 0.01

## Discussion

Gestational anemia is caused mainly by insufficient iron intake or depleted body stores. Pregnant women require 45 mg per day of iron, which is far greater than the non-pregnant requirement [2]. Therefore, iron replacement during pregnancy can avoid gestational anemia. As a result, iron supplementation during pregnancy can help prevent gestational anemia. In Iran, public prenatal care facilities provide free iron supplements to women, which explains why our study population has a lower prevalence of iron-deficiency anemia than other communities. Furthermore, as we can see, our people had good prenatal care access. However, because the small population diagnosed with iron-deficiency anemia may have adverse maternal and neonatal outcomes, we sought to assess the impact of iron-deficiency anemia on pregnancy outcomes. When the demographic characteristics of iron-deficiency anemic and non-iron-deficiency anemic women were compared, it was discovered that iron-deficiency anemia was more common in those with higher education. This is a novel finding, as previous research found that women with lower levels of education were more likely to suffer from anemia in both pregnant and non-pregnant populations [12]. Most studies linked lower education to lower socioeconomic status and insufficient access to prenatal care. However, we found that access to prenatal care was widespread in our cases. The explanation for the higher prevalence of anemia among those with higher education should be investigated using health behaviour theories, as some studies have suggested that individuals with higher education may perceive themselves to be at lower risk of developing certain diseases, causing them to avoid specific treatments or preventive health behaviours [13]. Among other demographic variables, living residency was linked to iron-deficiency anemia. Iron-deficiency anemic women mostly lived in rural areas, consistent with previous studies [13,14].

In terms of obstetrical factors, we observed that iron-deficiency anemic mothers had substantially more instrumental deliveries (all by vacuum) than non-iron-deficiency anemic mothers (4.3% vs. 0.8%), while the incidence of cesarean section was lower. This is a new finding as the previous systematic review showed that anemic women had a higher risk of cesarean [15]. However, the relation between gestational anemia and the mode of delivery remains unclear. More studies might lead to a better explanation. 

According to previous studies the incidence of preeclampsia and gestational diabetes are higher in iron-deficiency anemic women. Thus, iron-deficiency anemia may be a marker for other co-morbidities in pregnancy. A study by Ali et al. (2011) found that severe anemia during pregnancy can increase preeclampsia and eclampsia [16]. However, according to our findings, there were no differences in the rate of preeclampsia between iron-deficiency anemic and non-iron-deficiency anemic women. The association between gestational anemia and the frequency of gestational diabetes is controversial. According to the findings of a meta-analysis, the risk of gestational diabetes in pregnant women with iron deficiency anemia was less than in non-anemic women [17]. On the other hand, Kim et al. (2023) reported no association between the incidence of anemia and the risk of gestational diabetes [18], which aligns with our findings.

Regarding the impact of severe iron deficiency on the maternal outcome, we observed that iron-deficiency anemic women were at higher risk of postpartum hemorrhage. More blood transfusion was needed, which is rational. This is in accordance with previous studies [9,19]. The purpose behind the expanded risk of postpartum hemorrhage in anemic women is obscure. Yet, a few scientists accept that anemic women are more vulnerable to uterine atony due to weakened oxygen transport to the uterus [20,21].

Regarding neonatal outcomes, we discovered that iron-deficiency anemia increases the risk of low birth weight. This link is explained by the physiological fall in hemoglobin levels during pregnancy, which increases normal plasma volume and red cell mass [22]. The drop in maternal hemoglobin concentration may then impact fetal birth weight. This finding is consistent with other studies' findings [8,23]. This could be a result of intrauterine development limitation. The placenta expands and multiplies in an atmosphere devoid of oxygen. The fetal body experiences restricted oxygen circulation when the maternal hemoglobin level decreases. As a result, the fetal placenta is placed in an environment that causes oxidative stress. Intrauterine fetal hypoxia impedes the exchange of oxygen/supplement supply, limiting fetal development and low birth weight [24].

Low birth weight was another adverse neonatal outcome, according to our findings. Iron-deficiency anemic women were at higher risk of birth weight. This finding was in line with previous studies [11]. Iron deficiency increases oxidative damage to erythrocytes and the fetoplacental unit. Iron deficiency may also increase the risk of maternal infections, which can stimulate the production of corticotropin-releasing hormone and constitute a significant risk factor for preterm delivery [25]. Higher occurrence of low birth weight and preterm birth among anemic women are known factors associated with the increased risks of neonatal intensive care unit admission, as we observed in our study.

Our study suffers from some limitations that should be kept in mind in designing further research. First, although, from a clinical perspective, our study shows that poor maternal and neonatal outcomes occur more frequently in women with iron-deficiency anemia, as a retrospective cohort nature of a study, we cannot confirm that they are a straightforward consequence of iron-deficiency anemia. More interventional studies at an epidemiological level are desperately needed. Second, comparing the frequency with which iron supplements were taken between groups could have led to a better explanation, but we could not extract this information from patients' electronic charts. Further research considering the mentioned limitation is recommended. Third, separating low birth weight from preterm birth is another study's limitations. When gestational age is based on the last menstrual period, it is difficult and inaccurate to conclude low birth weight and preterm birth. Low birth weight strongly correlates with gestational age at delivery and other pregnancy complications like preeclampsia. Since there is no data on the gestational age at delivery or whether the preterm delivery was induced (as in preeclampsia/post-term) or spontaneous, it is difficult to conclude these complications. The other limitations of this study are the lack of comprehensive records, the quality of the documents, and recollection bias inherent in retrospective data collection. Lack of data in records of women who gave birth before 24 weeks of gestation and excluding these cases might affect the analysis. All these limitations should be considered. 

## Conclusions

Despite the higher incidence of postpartum hemorrhage and maternal blood transfusion, we found no increase in maternal intensive care unit admission risk in women with iron-deficiency anemia. Regarding neonatal outcomes, iron-deficiency anemia was linked to preterm birth, low birth weight, intrauterine growth retardation, and neonatal intensive care admission. Studies must be conducted to determine the value of treating anemia as an independent risk factor in predicting pregnancy outcomes.
